# Findings and lessons learnt implementing a cardiovascular disease quality improvement program in Australian primary care: a mixed method evaluation

**DOI:** 10.1186/s12913-021-07310-6

**Published:** 2022-01-26

**Authors:** C. M. Hespe, K. Giskes, M. F. Harris, D. Peiris

**Affiliations:** 1 School of Medicine, Sydney, University of Note Dame Australia, 160 Oxford St, Darlinghurst, Sydney, NSW 2010 Australia; 2 Heart Research Institute, University of Sydney, Sydney, Australia; 3 Centre for Primary Health Care and Equity, University of New South Wales, Sydney, Australia; 4 The George Institute for Global Health, University of Sydney, Sydney, Australia

**Keywords:** Quality improvement, Cardiovascular disease prevention, General practice, Quality improvement collaboration

## Abstract

**Background:**

There are discrepancies between evidence-based guidelines for screening and management of cardiovascular disease (CVD) and implementation in Australian general practice. Quality-improvement (QI) initiatives aim to reduce these gaps. This study evaluated a QI program (QPulse) that focussed on CVD assessment and management.

**Methods:**

This mixed-methods study explored the implementation of guidelines and adoption of a QI program with a CVD risk-reduction intervention in 34 general practices. CVD screening and management were measured pre- and post-intervention. Qualitative analyses examined participants’ Plan-Do-Study-Act (PDSA) goals and in-depth interviews with practice stakeholders focussed on barriers and enablers to the program and were analysed thematically using Normalisation Process Theory (NPT).

**Results:**

Pre- and post-intervention data were available from 15 practices (*n* = 19,562 and *n* = 20,249, respectively) and in-depth interviews from seven practices. At baseline, 45.0% of patients had their BMI measured and 15.6% had their waist circumference recorded in the past 2 years and blood pressure, lipids and smoking status were measured in 72.5, 61.5 and 65.3% of patients, respectively. Most high-risk patients (57.5%) were not prescribed risk-reducing medications. After the intervention there were no changes in the documentation and prevalence of risk factors, attainment of BP and lipid targets or prescription of CVD risk-reducing medications. However, there was variation in performance across practices with some showing isolated improvements, such as recording waist circumference (0.7-32.2% pre-intervention to 18.5-69.8% post-intervention), BMI and smoking assessment. Challenges to the program included: lack of time, need for technical support, a perceived lack of value for quality improvement work, difficulty disseminating knowledge across the practice team, tensions between the team and clinical staff and a part-time workforce.

**Conclusion:**

The barriers associated with this QI program was considerable in Australian GP practices. Findings highlighted they were not able to effectively operationalise the intervention due to numerous factors, ranging from lack of internal capacity and leadership to competing demands and insufficient external support.

**Trial registration:**

Australian New Zealand Clinical Trials Reference Number (ACTRN12615000108516), registered 06/02/2015.

**Supplementary Information:**

The online version contains supplementary material available at 10.1186/s12913-021-07310-6.

## Background

Cardiovascular diseases (CVD) are the leading cause of death worldwide, despite major declines in morbidity and mortality over the last 40 years [1]. In 2015, CVD was responsible for 29% of deaths, and over 1.1 million hospital admissions in Australia [2, 3]. CVD burden can be reduced through risk-factor modification [4]. Around two-thirds of Australians have three or more modifiable risk factors such as tobacco smoking, high blood pressure (BP) or cholesterol, physical inactivity, poor nutrition, or overweight/obesity [1, 5]. Most international guidelines recognise that these risk factors collectively contribute to an individual’s overall or ‘absolute’ risk, and that management decisions should be based on multiple risk factors [5–7]. In 2012, the National Vascular Disease Prevention Alliance launched Australia’s first absolute risk-based management guideline bringing together several guidelines into a single cohesive approach [5].

General Practitioners (GPs) play a major role in mitigating CVD morbidity and mortality, and see over 85% of the population (approx. 20 million consultations) in Australia annually [8]. However, studies have shown sub-optimal assessment and management of CVD risk. A 2006 study of The Bettering the Evaluation and Care of Health (BEACH) data found that patients at high risk of CVD were substantially under-treated [9]. In 2010, the AusHEART study found only 34% of patients at high CVD risk were prescribed both a BP-lowering medication and a statin [10] and with 2011/12 National Health Survey data Banks et al. showed that almost half (47.1%) of high-risk patients were not taking any guideline-recommended medications [4]. In 2012, baseline data from the TORPEDO randomised controlled trial found that only 48% of patients were appropriately screened for CVD risk [11], and similar findings were documented in a more recent review of Australian data from 2015 to 18 that showed 47.9% of patients had CVD risk screening and only 41% of high-risk patients were prescribed risk-reducing medications [12].

Quality Improvement (QI) initiatives in primary care have the potential to improve uptake of evidence-based practices [13]. QI is a multi-dimensional concept, which can be defined as having a systematic approach to making changes that will lead to better patient outcomes (health), enhanced system performance (care) and improved professional development (learning) [14]. There are several ways to intentionally implement QI initiatives, with one approach being the establishment of a Quality-Improvement Collaborative (QIC). QICs bring together groups of practitioners from different ‘organisations’ to learn about a specific aspect of health service quality, and to share experiences about making changes in their local settings. There has been mixed evidence of success implementing QICs in health care [15, 16]. However, a systematic review of 64 QIC programs in 2018 reported significant improvements in 83% of targeted clinical processes and patient outcomes [17].

The current study applied a QIC approach to improving CVD management in the ‘real world’ of Australian general practice in one Primary Health Network (PHN) in Sydney, Australia. Study aims were to: (1) assess whether a brief QIC program was associated with improvements in the monitoring, prescribing practices and attainment of BP and lipid targets for CVD risk reduction; and (2) understand barriers and factors driving implementation and adoption of the QIC.

## Methods

A mixed-methods sequential study design utilised data from the QPulse study (Central and Eastern Sydney General Practice Quality Improvement Network: building a sustainable model of QI to achieve reduced cardiovascular disease in the primary care setting) [12, 18]. The program was developed by the research team who had extensive involvement and collaboration with the PHN with a number of programs over the past decade. The QIC was designed to be overseen and coordinated by CESPHN to complement previous quality improvement programs and utilise existing relationships between PHN staff and the general practices. Prior to commencement, the study protocol was approved and registered by the Australian and New Zealand Clinical Trials Registry (https://www.anzctr.org.au/Trial/Registration/TrialReview.aspx?ACTRN=12615000108516). A protocol and set of QI resources were developed for the PHN to implement the program.

A QPulse program officer was employed by the PHN. She worked as part of the PHN QI team who oversaw the other QI projects within their footprint. All PHN staff working on QPulse received standardised training from the research group in the CVD risk-reduction intervention, the QI program and the implementation processes. The QI team facilitated the workshops and oversaw the Plan Do Study Act (PDSA) activity. There were regular program updates and meetings with both PHN staff and the research group to support the program roll-out, including discussion around recruitment and implementation. Similarly, there were regular meetings between the lead researcher and program officers to discuss issues and difficulties with the intervention and program implementation.

### General practice recruitment

**Fig. 1:**
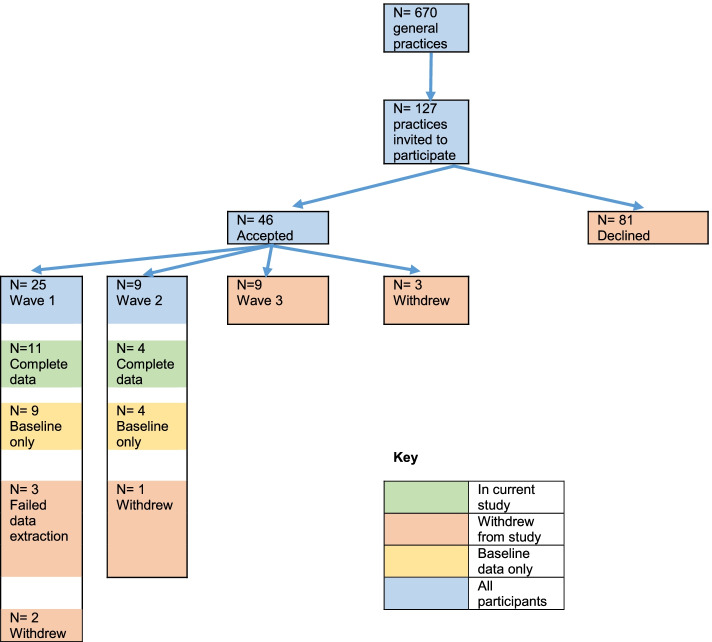
Practice Recruitment to the QPulse study

Practices were recruited from the geographical catchment of Central and Eastern Sydney Primary Health Network (Australia) between May 2015 and November 2016. Practice recruitment is outlined in Fig. 1. PHNs are federally-funded meso-tier organisations tasked with supporting the primary health care system, commissioning of services, and working collaboratively to integrate health services within their region. A number of methods were employed to recruit practices, including targeting those that had participated in other QI programs, informal invitation at professional development or local network meetings, and formal invitation via email, newsletters and/or weekly fax communiques to GPs and practice managers (PM).

Practices were eligible if they used one of two electronic medical record software programs (i.e. Medical Director™ or Best Practice™) for recording risk-factor information, pathology results and prescriptions. There was a total of 127 eligible general practices from a possible 670 in the CESPHN catchment that were approached to participate. Reasons given to the PHN team from 41 of invited non-participating practices included: not enough time, staffing limitations, competing priorities, CVD not being a practice priority area, and not wanting to participate in research.

There were three waves of recruitment between April 2015 - July 2017; 25 practices were included in Wave 1, nine in Wave 2 and nine in Wave 3. Wave 3 was subsequently cancelled due to lack of PHN staff resources, and consequently only practices from Waves 1 and 2 (*n* = 34) participated, with three withdrawing. Practice participation rates were lower than anticipated, and the study did not achieve the target recruitment of 80 practices prior to cessation of recruitment. Recruitment ceased after the PHN felt unable to provide resources to the program, in November 2017. Complete pre- and post-intervention data were available from 15 GP practices. In 16 practices complete data-sets were not successfully extracted using the data extraction software due to a technical problem in the extraction of prescribed medications from some of the practice records, and issues in the automated delivery of the extracted data to the secure study portal (see Fig. 1). These software problems resulted in baseline data only being available for 13 of the participating practices (9 practices in wave 1, 4 practices in wave 2). Data extraction failed for both baseline and follow-up data extractions in 3 Wave 1 practices. Practices included in the study covered the geographic area of the PHN, represented practices with different billing structures (i.e. bulk- and private-billing) and included both independent- and corporate-owned practices.

The baseline patient characteristics of the 19 services with only pre-intervention data were similar to the 15 with complete (i.e. pre- and post-intervention) data (Supplementary Table 1) that have been included in this study. There were no differences in baseline patient demographic, CVD assessment and risk factor profiles between the two samples.

### Quality improvement program

The QPulse program ran over 22 months (May 2015 – February 2017). Participating practices provided the PHN with baseline, de-identified patient data of ‘regularly attending’ patients (defined below). The PHN generated feedback reports at the commencement of the intervention (using baseline data) for each practice targeting the CVD measurable goals.

At least one stakeholder from each practice, such as a practice nurse, senior GP or PM attended three 2-h workshops over 3 weeks. Topics covered: CVD risk assessment tools, absolute risk calculators, current guidelines for preventive CVD management including pharmacological management of high-risk patients, QI theory and specifics on how to achieve change using the Plan Do Study Act (PDSA) methodology [19]. By planning a change, trying it, observing the results, and acting on what was learned, participants were guided through a rapid feedback exercise to generate change ideas for their practice. Depending on their specific aim, practice teams were asked to choose ideas to test on a small scale and refine the change as necessary before implementing successful ideas more broadly. Each practice team was asked to submit a monthly PDSA report to the PHN to assist them in planning and measuring change.

During the workshops, participants were also trained in the use of two quality-improvement software tools supplied to each practice: (1) a desktop ‘point of care’ tool, ‘HealthTracker’; a software application that works in conjunction with the electronic medical record to identify high-risk patients and provides GPs with ‘real time’ personalised guideline recommendations pertaining to cardiovascular risk reduction; and (2) PenCAT Clinical Audit Tool™ a data extraction tool installed onto the computer server at each Practice. PenCAT enabled practices to download specific audit reports containing de-identified patient data on demographics, CVD disease statistics and prescribing information. A data extraction could be performed as frequently as practice staff desired to assist with the PDSA process and was the basis of the monthly data report sent to the PHN.

Members of each general practice were also invited to attend monthly webinars after the initial three workshops to collaboratively share their progress and ideas for change with other participating practice teams. Each practice was offered external support from the PHN to assist with provision and interpretation of the personalised practice report, formulating the PDSAs, and submitting monthly data extractions.

### Target population

Eligible patients were those recommended by guidelines for CVD risk assessment [5], and who were ‘regular attenders’ at the practice. This included Aboriginal and Torres Strait Islander people ≥35 years and all others ≥45 years; and those at clinically high risk of CVD regardless of age (defined below). ‘Regular attenders’ were defined as patients who attended the practice at least three times in the previous 24 months, and at least once in the previous 6-month period.

Five-year CVD risk was calculated using the 1991 Anderson Framingham Risk equation using sex, age, systolic blood pressure, smoking status, total cholesterol, high density lipoprotein (HDL) cholesterol and diabetes status [20]. Diabetes and left ventricular hypertrophy were assumed to be absent, unless explicitly recorded as diagnoses in the patient record. As per current guidelines, high CVD risk was defined as any of the following: (i) calculated 5-year CVD risk exceeding 15% based on the FRE, (ii) presence of clinically high-risk conditions (including diabetes and age > 60 years, diabetes and albuminuria, eGFR < 45 ml/min/1.73m^2^, systolic BP > 180 mmHg, diastolic BP > 110 mmHg or total cholesterol > 7.5 mmol/L) (iii) presence of a CVD diagnosis (i.e. coronary heart disease, cerebrovascular disease, peripheral vascular disease) [5].

### Outcome measures

Outcome measures were: (1) CVD risk factor assessment including BP assessment in the past year, and lipid, BMI, waist circumference and smoking status assessment in the past 2 years; (2) guideline-recommended treatment for people defined as prescription of a BP-lowering medication and a statin for people at high CVD risk and prescription of a BP-lowering medication, a statin and either an antiplatelet or an anticoagulant agent for people with a diagnosis of existing CVD (see Appendix 1 for specific medications); and (3) meeting CVD targets, which were defined as a BP less than 140/90 mmHg for high-risk patients, and less than 130/80 mmHg for those with established CVD or diabetes and a total cholesterol < 4.0 mmol/L, high-density lipoprotein (HDL) level of > 1.0 mmol/L, low-density lipoprotein (LDL) level of < 2.0 mmol/L.

### Statistical analyses

Bivariate analyses examined the frequencies and proportions of patients by their sociodemographic, CVD risk, BP/lipid levels and prescription of risk-reducing medications. Logistic regression models examined differences between baseline and post-intervention in the key outcome variables with adjustment for gender, age and Indigenous status. Changes between baseline and post-intervention were assessed by odds ratios (95% CIs), using the baseline sample as the reference category. As the data were hierarchical (i.e. patients clustered within GP practices), all analyses were adjusted for clustering at the practice level and applying finite population correction when estimating variance. Analyses were conducted with SPSS statistics software Version 26.0.0. Statistical significance was considered as *p* < 0.05 (two-tailed).

### Qualitative data analysis

The PDSAs submitted by each practice were analysed by thematic analysis. The goals addressed in the PDSAs were coded by two coders who worked independently. Coders then sorted the themes of the PDSAs into larger categories and then discussed their results, examined any discrepancies and reached consensus for a final coding classification. Following this, 19 semi-structured interviews were conducted after completion of the program with a purposive sample of people involved in QPulse implementation, including PMs, nurses and GPs, as well as program officers, IT support personnel and managers at the PHN (Table 1). The interview guide is provided in Tables 4 and 5, Appendix 2.

**Table 1:** Interviewee characteristics from the Primary Health Networks (PHNs) and from general practices

**Interview participants from PHNs (*n*** **= 7)**	
Female	4
Program Officer	2
Team Manager	2
Executive Officer	1
IT Support Officer	2
**Interview participants from general practices (*n*** **= 12)**	
Female	9
Practice nurse	1
Practice manager	1
General Practitioner	10
Practice size (number of regular patients)	
< 2000	1
2001- 4000	1
4001-6000	3
6001-8000	3
8001-10,000	2
10,001-20,000	1
> 20,001	1
Previous QI experience	6
No previous QI experience	6

Interviews were digitally recorded and professionally transcribed. Transcripts were shared with the interviewees to ensure they were accurate, and that they were agreeable to the contents being used for the study. Four researchers independently read and analysed the interview transcripts; this included the principal investigator and three researchers who had not taken part in the QPulse study. Interviews were manually coded by each researcher guided by the domains in Normalisation Process Theory (NPT) [21]. The findings were iteratively reviewed and refined at two group meetings with the principal investigator and three researchers.

NPT is a theory of implementation designed to aid interpretation of how interventions are embedded, enacted and operationalised within routine practice in healthcare settings. This approach assumes four main generative mechanisms (coherence or sense making; cognitive participation, collective actions; and reflexive monitoring) which are needed to achieve change in practice. The aim of the analysis was to examine the ‘real world’ participant perceptions of, and responses to the multicomponent QI program, and at which points it was considered to be sustainable or have failed [21].

This research was performed in accordance with the Declaration of Helsinki and was approved by the University of Notre Dame Australia Human Research Ethics Committee (HREC) (reference 014105S). Signed agreements with participating practices were also obtained. A consent waiver for patient-level consent was granted by the committee.

## Results

### Practice characteristics and engagement

Of the 15 practices with complete pre- and post-data, all attended at least one workshop, 11 attending two and six attended all three workshops. Most practices sent only one attendee to each workshop with three practices sending two attendees and one practice sending three attendees to all three workshops. The PHN recorded contact with all participating practices at least once a month, some requested higher levels of interaction (range 1 to 8 contacts per month) which was provided via phone or face-to-face, to help with IT and QI processes. The program officer recorded a median of four practice visits and 15 phone calls per practice over the duration of the program. Although 12 practices registered to attend the first two webinars, only two attended, and these were consequently discontinued after 2 months. All participating practices submitted a baseline PDSA.

### Baseline patient characteristics

The mean age of patients at baseline was 63.9 years, and the majority (55.3%, *n* = 10,816) were female. 5.8% (*n* = 1139) had high absolute cardiovascular disease or high clinical risk, and 12.1% (*n* = 2372) already had a diagnosis of CVD (Table 2). A greater proportion of patients at high risk of CVD or established CVD had up-to-date BP, lipid, BMI and waist circumference measures than those with low/moderate risk. High-risk patients were more likely to be overweight/obese or current smokers than their low/moderate risk counterparts. They were also more likely to reach total cholesterol and LDL targets, however a lower proportion met BP and HDL targets. A minority (41.2%) of high-risk patients were prescribed recommended medication, whereas a majority (69.5%) of those with established CVD were prescribed the recommended risk-reducing medications.

**Table 2:** Pre- and post-intervention data of sociodemographic, cardiovascular risk assessment and management in QPULSE

	Pre-intervention				Post-intervention			
	Cardiovascular risk assessment			Total sample in 15 practices (n = 19,562)	Cardiovascular risk assessment			Total sample in 15 practices (***n*** = 20,249)
	Low/moderate (***n*** = 8171)	High (n = 1139)	Established CVD (***n*** = 2372)		Low/moderate (***n*** = 9155)	High (***n*** = 1195)	Established CVD (***n*** = 2537)	
**Age (mean, SD)**	61.1 (11.2)	72.4 (9.2)	73.6 (11.3)	63.9 (12.5)	60.8 (11.1)	71.9 (9.2)	73.5 (11.4)	63.8 (12.4)
**Gender**								
Male	38.2 (3124)	78.7 (896)	62.9 (1491)	44.6 (8717)	38.5 (3525)	78.4 (937)	61.8 (1569)	55.5 (11236)
Female	61.8 (5047)	21.3 (243)	37.1 (879)	55.3 (10816)	61.5 (5630)	21.6 (258)	38.1 (967)	44.5 (9013)
Missing				0.1 (29)				0.1 (13)
**Ethnicity**								
Aboriginal or Torres Strait Islander	0.4 (30)	0.5 (6)	0.3 (6)	0.4 (77)	0.4 (35)	0.7 (8)	0.5 (12)	0.5 (109)
Other	99.6 (8141)	99.5 (1133)	99.7 (2366)	99.6 (19485)	99.6 (9120)	99.3 (1187)	99.5 (2525)	99.5 (20153)
**Risk factor assessment**								
Blood pressure^a^	81.9 (6690)	86.1 (981)	81.0 (1922)	72.5 (14175)	85.9 (7862)	93.0 (1111)	83.5 (2119)	76.4 (15487)
Blood lipids^b^	82.5 (6739)	82.0 (934)	70.7 (1678)	61.5 (12026)	85.0 (7781)	86.5 (1034)	75.5 (1916)	65.9 (13361)
BMI^b^	54.6 (4460)	57.4 (654)	49.4 (1171)	45.0 (8814)	59.0 (5394)	65.4 (782)	45.1 (1347)	49.4 (10000)
Waist circumference^b^	21.0 (1719)	25.3 (288)	15.9 (377)	15.6 (3039)	25.3 (2314)	32.4 (387)	19.9 (506)	34.7 (3952)
Smoking status	63.2 (5164)	70.1 (798)	71.1 (1686)	65.3 (12774)	63.9 (5785)	65.3 (780)	66.8 (1695)	65.7 (13304)
**BMI**								
Underweight	3.2 (260)	1.1 (13)	2.1 (51)	2.5 (484)	3.4 (310)	0.8 (10)	2.6 (65)	2.7 (541)
Healthy weight	21.6 (1768)	13.6 (155)	14.2 (336)	16.8 (3282)	23.1 (2114)	15.8 (189)	15.2 (386)	18.1 (3675)
Overweight/obese	26.8 (2189)	34.3 (391)	26.9 (639)	22.8 (4467)	27.3 (2503)	31.5 (377)	27.9 (707)	23.6 (4786)
Not assessed/missing	48.4 (3954)	50.9 (580)	56.7 (1346)	57.9 (11329)	46.2 (4228)	51.8 (619)	54.4 (1379)	55.6 (11260)
**Waist circumference**								
Normal	9.1 (741)	7.0 (80)	5.1 (122)	6.4 (1250)	11.4 (1047)	9.5 (113)	5.8 (148)	8.0 (1619)
At risk^c^	22.5 (1840)	31.0 (353)	22.8 (541)	18.1 (3546)	26.2 (2398)	33.6 (402)	26.3 (666)	21.4 (4330)
Not assessed/missing	68.4 (5590)	62.0 (706)	72.0 (1709)	75.5 (14766)	62.4 (5710)	56.9 (680)	67.9 (1723)	70.6 (14313)
**Smoking status**								
Never smoker	67.7 (5532)	43.3 (494)	48.8 (1157)	55.9 (10942)	68.4 (6260)	42.0 (502)	49.0 (1242)	57.6 (11673)
Current smoker	22.1 (1808)	25.1 (286)	31.5 (747)	20.3 (3978)	22.2 (2028)	24.5 (293)	31.1 (789)	20.5 (4155)
Ex-smoker	10.2 (831)	31.5 (359)	9.3 (220)	10.9 (2130)	9.5 (867)	33.5 (400)	9.8 (249)	10.9 (2211)
Missing			10.5 (248)	12.8 (2512)			10.1 (257)	11.0 (2223)
**CVD targets achieved**								
Blood pressure	79.6 (5325)	20.7 (203)	42.2 (811)	67.8 (9609)	80.9 (6360)	20.5 (228)	46.1 (976)	68.4 (10586)
Total cholesterol	11.4 (769)	22.7 (212)	53.9 (904)	20.5 (2462)	11.3 (877)	24.7 (255)	52.5 (1006)	20.3 (2708)
LDL cholesterol	13.9 (938)	26.0 (243)	55.2 (926)	25.0 (3010)	13.9 (1080)	25.8 (267)	54.5 (1043)	24.6 (3282)
HDL cholesterol	92.5 (6234)	73.4 (686)	78.7 (1320)	86.7 (10418)	93.0 (7240)	73.2 (757)	80.1 (1534)	86.6 (11698)
**Prescribed BP- and lipid-lowering medications**^**d**^	16.6 (1357)	42.5 (484)	69.8 (1656)	25.1 (4901)	16.0 (1464)	44.1 (527)	68.9 (1748)	25.2 (5096)

^a^Assessed in the past 12 months

^b^Assessed in the past 2 years

^c^‘At risk’ waist circumference > 94 cm for males and > 80 cm for females

^d^Guideline-recommended treatment was defined as [1] For high-risk patients the prescription of a BP-lowering medication and a statin [5]; for patients with established CVD, prescription of a BP-lowering medication, a statin and either an antiplatelet or an anticoagulant agent (see Appendix 1 for specific medications)

### Post- intervention

The sociodemographic profile of patients in the post-intervention sample was similar to the baseline characteristics, as were the CVD risk factor profiles, screening and management (Table 2). Post-intervention cardiovascular risk assessment, risk profiles and CVD targets were compared with baseline in Table 3. There were no significant improvements in any of the outcomes post-intervention, however there was wide variation in practice performance. Supplementary Table 2 provides summaries of the results of four high-performing practices where marked changes in selected outcomes were observed and showed improvements in the recording of BMI in Practices 1 and 2 after the intervention. Practices 1-3 also showed large improvements in the measurement of waist circumference, whereas Practice 4 had comparatively high baseline levels of all risk factor assessment and showed no overall change in these measures post-intervention.

**Table 3:** Changes in CVD risk factor assessment, CVD targets and prescribing behaviours post-intervention compared to baseline

	OR (95% CI) post-intervention compared to baseline			
	Total post-intervention sample in 15 practices (***n*** = 40,256)	Cardiovascular risk assessment level post-intervention		
		Low/moderate (***n*** = 17,365)	High (***n*** = 2334)	Established CVD (***n*** = 4975)
**Risk factor assessment**				
Blood pressure^a^	1.23 (0.90-1.70)	1.36 (0.82-2.25)	1.12 (0.85-1.48)	1.22 (0.80-1.86)
Blood lipids^b^	1.22 (0.94-1.58)	1.21 (0.75-1.95)	1.40 (0.56-3.49)	1.29 (0.93-1.79)
BMI^b^	1.19 (0.99-1.42)	1.19 (0.95-1.50)	1.40 (0.90-2.19)	1.17 (0.93-1.46)
Waist^b^ circumference^b^	1.32 (0.74-2.35)	1.27 (0.61-2.65)	1.41 (0.81-2.45)	1.34 (0.88-2.03)
Smoking status^b^	1.17 (0.91-1.48)	1.13 (0.91-1.38)	1.09 (0.75-1.38)	1.11 (0.88-1.38)
**CVD risk factors**				
Overweight/obesity	0.97 (0.94-1.00)	0.96 (0.91-1.01)	0.80 (0.63-1.02)	1.00 (−.90-1.11)
Waist circumference^c^	1.02 (0.68- 1.53)	0.99 (0.62-1.60)	0.86 (0.61-1.22)	1.07 (0.73-1.55)
Current smoker	0.99 (0.94- 1.05)	0.91 (0.81-1.02)	1.05 (0.98-1.13)	1.05 (0.92-1.20)
**CVD targets achieved**				
Blood pressure	1.02 (0.90-1.16)	1.08 (0.97-1.21)	0.99 (0.70-1.40)	1.16 (1.02-1.33)
Total cholesterol	0.99 (0.92-1.06)	0.99 (0.91-1.08)	1.15 (0.90-1.46)	0.95 (0.84-1.07)
LDL cholesterol	0.98 (0.92-1.04)	1.00 (0.94-1.06)	1.00 (0.83-1.20)	0.98 (0.87-1.11)
HDL cholesterol	1.08 (0.99-1.18)	1.09 (1.00-1.20)	0.99 (0.84-1.13)	1.08 (0.95-1.23)
**Prescribed CVD risk-reducing medications**				
Prescribed risk-reducing medication(s)^d^	–	–	1.07 (0.97-1.19)	0.96 (0.91-1.02)

^a^Assessed in the past 12 months

^b^Assessed in the past 2 years

^c^‘At risk’ waist circumference > 94 cm for males and > 80 cm for females

^d^Guideline-recommended treatment was defined as [1] For high-risk patients the prescription of a BP-lowering medication and a statin [5]; for patients with established CVD, prescription of a BP-lowering medication, a statin and either an antiplatelet or an anticoagulant agent (see Appendix 1 for specific medications)

### PDSA themes

**Fig. 2:**
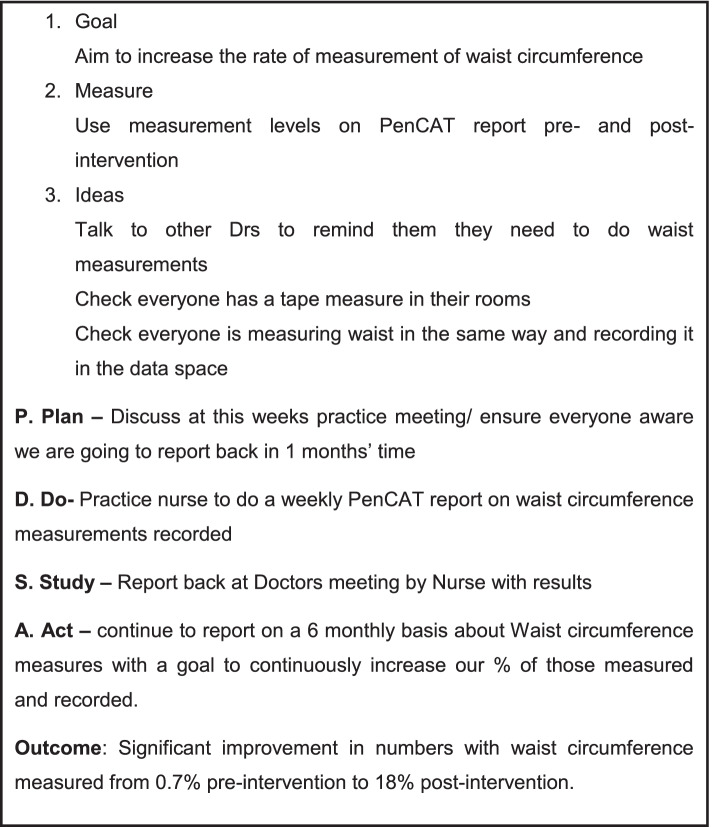
Plan-Do-Study-Act (PDSA) exemplar from one practice

Every practice submitted at least one PDSA with only one practice submitting monthly, as requested. Overall, the PDSAs had an emphasis on foundational goals such as improving data measurements (e.g. recording of waist circumference or smoking status) rather than focusing on changes in preventive care and guideline-based prescribing (neither lifestyle nor medication). In some cases, PDSA goals aligned with improvements in the practice recording risk factor data, as seen in one practice (Fig. 2) that focused on waist circumference measurement.

### Interview analysis

The characteristics of interview participants at the PHN and practice levels are shown in Table 1. There were a range of stakeholders interviewed within various roles and levels of the organisations, with a good distribution of participants in practices are varying size and with prior QI experience. The interviews yielded rich insights into understanding the quantitative findings. Multiple barriers to implementation across all four NPT domains were described and summarised below.

#### Coherence (the meaning ascribed individually and collectively to a new set of practices)

Collectively, GPs and PHN staff saw the potential value of the program as very high, for example: “*There are a whole lot of people at risk, and they could have better outcomes….”* (GP6). Access to the education modules and the two QI tools were seen as useful in identifying and providing preventive care guidelines to high-risk patients. Only one participant mentioned that internal leadership (‘Change Champion’) or practice support (‘QI Culture’) was needed to facilitate engagement with the program.

#### Cognitive participation (commitment to engage with the new)

Interviewees from both PHNs and practices noted that the timing of this program (coinciding with major PHN governance and strategy change) prohibited prioritisation of support for the program by PHN Staff.

Most interviewees reported they had not fully appreciated what participation in a QI program would require of them prior to the program, *“…when I took it on I didn’t realise there was more to it, so I didn’t really understand...*”. (GP1). For PHN participants this was illustrated at the highest executive level where management under-estimated the readiness/ability of practices to be enrolled in this program, along with the need for the PHN to supply adequate resourcing to support both their staff and the participant practices. “*With 40 practices enrolled, the workload sometimes got overwhelming despite team members help(ing) me with 6 of the practices*”. (PHN3) This lack of strategic planning and resourcing was amplified by the change in focus of PHN staff and a merger of three earlier meso-tier organisations into a single PHN. This affected directly on the PHN’s ability and commitment to supply practice-level support during the program.

GP engagement with the PDSA process was also extremely low. While the PDSAs were understood by most GPs as an essential part of the QI process, they were seen as time-consuming, and “*…. formulaic…. uninteresting…*” (GP1) and *“...to be honest, no, I haven’t done one since we started*.” (GP9) with only a few seeing value in this aspect of the program. “*It is a problem to stay on track and keep getting things done… I had a million good intentions, and then it gets …too hard*”. (P4)

#### Collective action (how the work does/not get done)

Participants reported a lack of both ‘QI culture’ and change champions to support engagement with QPulse. Although there was universal agreement that ‘key individuals were needed to drive the program forward’, this did not mean that these individuals were found, nor engaged to help with the program. “…*it comes down to the culture within the practice, who is the real leader…the driver in the practice. It could be a nurse or the doctor…. but crucially you really need to have somebody who is going to take the reins, or it doesn’t happen…*.” (PHN5). It was clear many participants were unable to actively champion or drive the program forward due to not taking on a leadership role within their practice setting. Differing practice systems often meant each GP within the practice worked as an individual rather than part of a cohesive system of care. “*It’s quite individual. That’s the way the practice is set up…. your quality control is up to you…. As long as it doesn’t add any extra work…. because no-one obviously is interested if it’s extra work*.” (GP2)

Communication systems between GPs, nurses and PMs were cited as a barrier to engagement. Often there was no regular practice ‘team’ meetings or systems in place to report back about QI measures and limited ability to organise tailored educational activities. Jobs were delegated to non-medical staff (e.g. the PM or nurse) who may/not have the skills or motivation to drive the program forward due to a lack of personal engagement with the goals of the program. At one practice, a manager commented that she only became involved because: (the GP) *“…didn’t want to do it, so she handed it to me… I actually didn’t know much about it.”* (GP12). In addition, GP attendance at the educational sessions was sub-optimal and inconsistent, with many GPs delegating this to the PM or nurse. The scheduled monthly networking/seminar meetings were cancelled due to lack of attendance, despite most participants opting to join at the beginning of the program.

There was commentary that the QI program and data reports did not address key issues around improving engagement with patients: *“It’s one thing to get the GPs to change what they do, it’s an entirely different thing to get patients to take in on board.” (GP 6)* Several GPs also discussed the problem with many competing projects and lack of time: “*I think the difficulty is there’s a plan for one quality of project and then another idea comes up and then the same people are looking at implementing it, or we give the nurses something else to do, and it sort of falls off the radar*.” (GP 6)

Participants reported barriers to setting up a sustainable QI processes. This was highlighted by commentary about the difficulty of scheduling data extractions and generation of reports, low attendance at QI educational activities, no engagement with PDSA process, no evidence of sustained use of the IT tools. Several attributed this to both lack of dedicated time to do QI work and lack of any tangible incentives (financial or professional development). Many reported that GPs were not keen to engage with an activity that was not aligned with financial incentives and cited such incentives as a mechanism to achieve long-term engagement rather than as the first reason for engagement. However, engagement with individual “contracted” GPs was reported as requiring a financial incentive to engage them with doing any of the extra work involved in QI activities. “…*from a practice perspective, it’s not going to be a priority. So really, the PHN needs to take on a lot of that responsibility on behalf of the practice if we’re going to get it up and running*”. (PHN1)

Minor IT issues were also identified as barriers, although it was well understood by participants that IT support was readily available if asked: “*I used it for a couple of weeks and found it was really useful... (when a minor IT issue arose to render the tool inactive) …it just kind of died off, my use of it*.” (GP7) Finally, it was noted that the program did not adequately accommodate the roles of individual GPs (and patients) in achieving key outcomes (such as BP, lipids levels) and medication prescriptions. *“It was all a bit clunky…I never saw the Manager’s data reports…and although I tried with Q Pulse, …if a doctor is not interested, they won’t do it ...that’s often the way around here and I just couldn’t engage them with recording the CVD measures*.” (GP2)

PHN staff reported a lack of resourcing to provide individualised GP practice reports, education or face-to-face support despite observing that most practices needed significantly more support than anticipated to complete the basic requirements of the program (such as monthly data extractions, PDSAs, tidying up eMR systems). “*Until you start giving monthly reports and with targeted topics and actually educating the GPs about what to do with that data, you’re just extracting data*”. (PHN1)

#### Reflexive monitoring (the processes through which practitioners decide whether new approaches are beneficial and lends, ultimately, to the normalisation of new practices)

Normalisation of systematised QI practices was not apparent, even among the most experienced and engaged practices. This was despite many participants reporting enthusiasm for ongoing participation in QI work. “*So, we’ve talked about it, but we’ve never implemented it systematically…. most doctors are not taught these things, that’s the whole problem, so we’ve got to re-educate the doctors, our universities haven’t got it in the curriculum*.” (GP7)

Even in solo practices, GPs experienced challenges with translating the QI goals into long-term changes systematic CVD preventive care. The program learnings were discussed as a one-off piece of work rather than something to embed into everyday routine practice. *“It’s the follow through which can be difficult because you will forget. I don’t know that this is common to everyone, but ...if somebody is not ...pestering you and reminding you... - I’ve actually forgotten how to do it - it falls off the wagon.”* (GP 3)

Some GPs reported adopting new ways of approaching CVD preventive care, such as utilising CVD absolute risk assessment tools or using audit data extraction tools to identify gaps in data measures such as smoking status, BMI or waist circumference, “*every single patient I see I just flick onto the summary screen of HealthTracker and just see whether the percentage is something I need to worry about, and if it’s not, then I don’t pursue it.”* (GP 4) However, few mentioned systematic use of the tools for the whole team. Even the most engaged general practice participants noted a difficulty in systematising QI in the day-to-day running of business. *“I think (the workplace of) general practice is a barrier, it’s an unpredictable, busy, chaotic job and so things happen that get in the way. And I think the other barrier is that protected time, to set up systems is an enabler and not being in place is a barrier*”. (GP 6)

## Discussion

Engagement with the QPulse QI program was limited in most general practices. Pre-intervention data showed a sub-optimal assessment of CVD risk factors, along with low prescribing rates among high-risk patients. After the program there were no significant changes in the assessment of CVD risk factors, the achievement of BP and blood lipid targets or the prescription of risk-reducing medications. Normalisation Process theory provided a framework to analyse participants experiences of implementing the program and focused on the work required to embed and normalise QI processes in the general practice settings [22]. The qualitative data demonstrated significant real-world barriers in implementation in all four NPT domains but particularly within collective action and reflexive monitoring. The framework assisted in demonstrating specific areas where future implementation strategies should be emphasised.

Overall, GPs considered the program as relevant and beneficial to patient care. Stakeholders identified that activity was mainly led by non-medical staff and focussed on improving documentation rather than clinical outcomes. There were substantial challenges for GPs for embedding the program into long-term changes in clinical practice. It is difficult to ascertain whether these were due to limitations in the design of the program or the intervention, or in its delivery, however given the limited success of many interventions in primary care [11, 23, 24] it is likely that implementation played a larger role. Primary health care is a complex environment that benefits from structured systems of care to aid the adoption of best practice. The privatised model of Australian general practice, which rewards clinicians for volume-based care is a financial and philosophical barrier to widespread adoption of QI programs which focus on ‘value- based’ care [18].

### Lessons learned about quality improvement collaborations in Australian general practice

The challenges associated with achieving collective action for both general practices and the PHN meant participants found it difficult to move beyond ‘new’ and toward ‘normal’. Interviewees reported a ‘project-based’ approach to QI with ‘topical’ engagement and difficulty setting up systematised adoption of change due to the considerable number of competing projects. At the time of this program, there was no systematic funding or incentives for GPs and practices to improve their data collection processes, and such improvements were mainly driven by practice ‘champions’ and needed to be supported by the systems and IT infrastructure to normalise these changes. These supports were not in place and were identified as key factors limiting the program in the stakeholder interviews. In many cases, there was no clear role of a leader within practices, and when leadership developed it was often from non-medical staff. Furthermore, this QI program was unable to capture doctor-patient decision making around individual choices and the adoption of CVD risk recommendations, including medications, diet and exercise.

The findings also suggest that implementing change needs to build practice capacity and culture; change needs to be supported by GPs, practice managers and reception staff, and the necessary IT systems, staffing and time allocation to QI activities need to be made available. The results showed that even after the QI program, a substantial proportion of practices (30-40%) were not meeting basic documentation and monitoring targets. These improvements have been identified as key to increasing uptake of evidence-based practice in primary care [13, 16]. This study highlights the need for concerted efforts to set up these foundations at the individual GP, practice and PHN levels to improve the uptake of QI programs.

Large-scale QI research in primary care in the US has concluded that practice support is essential to help practices, particularly those with electronic medical record data challenges, build their capacity for conducting data-driven QI [25]. Data is a fundamental step in participating in service improvements in primary care, and for performance-based payment programs [26]. A qualitative review of QI programs in Australia similarly identified the need for external support, accurate data and reporting, education and change champions in addition to financial incentives [18]. Government-funded initiatives such as heart health assessments, health assessments for 45–49-year-olds, GP management plans and home medication reviews have only had partial uptake and mixed evidence of success [8, 27, 28]. One potential driver of their mixed success is that they are still fee-for-service activities and do not support GPs to adopt quality-focused models of care [29, 30]. A recent evaluation of the Health Care Home initiative in Australia, an initiative for adopting a patient-centred model of care for those with chronic and complex health conditions, identified they key role of practice facilitators with advanced facilitation skills for implementing change in general practices [31].

While there has been increased interest in supporting QI in Australian primary care, there needs to be a change in thinking from start-up programs to sustained initiatives. A pre-post evaluation of a national QI program, the Australian Primary Care Collaborative program from 2004 to 2014, found improvements in both CVD risk identification and management, but only had partial uptake nationally [13] and did not receive ongoing Federal funding. Driving this program relied on a complex interaction of enablers such as leadership, accurate data, funding incentives, organisational culture, and primary care systems designed to support quality care [18, 23, 32]. Interim evaluation of the Health Care Home Initiative in Australia also supported allowing for sufficient time for the implementation of complex programs in primary care [31]. Scoping the focus of the program to addressing very targeted outcomes rather than implementing broader more complex QI initiatives has also been a recommendation from a large trial evaluation of the EvidenceNOW model in the US [33].

### Study limitations

The current study had a number of limitations that need to be acknowledged in the context of the results. Firstly the practices were located in one PHN region and may not be representative of general practices elsewhere. For example, we have previously found higher performance for CVD risk management in Aboriginal Community Controlled Health Services compared with other general practices [10]. Secondly, although de-identified electronic medical record data extraction tools allow for a ‘whole-of-practice’ snapshot of performance, the data need to be entered in extractable fields of the record. This may result in an underestimate of management practices [34]. This mainly applies to BP and smoking status, given that electronic prescribing and pathology results are adopted in most Australian general practices. Thirdly, in assessing measurement of absolute CVD risk we looked at the recording of risk factor data but were not able to assess to what extent risk scores were calculated. Although HealthTracker, the point of care decision tool supplied to each practice, did provide this information for each patient at the time of consultation, we did not have user analytics on whether an absolute risk score was generated. Given GPs vary greatly in how they use risk scores, the gaps in risk-based management may also vary when using surrogate measures to capture the outcomes of complex GP-patient interactions [35]. Furthermore, the lower-than anticipated practice numbers resulted in some effect sizes being non-significant.

## Conclusions

There are large gaps between CVD management guidelines and practice in primary care in Australia. This QI program targeting CVD risk identification and management did not bring about overall changes in risk factor documentation, risk factor prevalence, attainment of physiological targets and prescribing for risk reduction. Overall, practitioners see the value in QI programs targeting CVD, however experience substantial challenges in implementing change if this is not supported more widely. GPs and other practice staff need support at all levels of the organisation for QI, in addition to the time, technical support and appropriate remuneration to facilitate long-term improvements in the management of CVD in general practice.

### Supplementary Information

**Additional file 1: Supplementary Table 1.** Baseline characteristics of included and excluded patients. **Supplementary Table 2.** Pre- and post-intervention characteristics of 4 sample practices.

## Data Availability

The datasets generated and/or analysed during the current study are not publicly available but data may be made available to interested parties from the corresponding author on reasonable request.

## Appendix 1

### Medications list

A complete list of the medicine active ingredients audited in this study

#### Anti-coagulant medications

**Table:**

APIXABAN	
BIVALIRUDIN	
DABIGATRAN	
DALTEPARIN	
ENOXAPARIN	
FONDAPARINUX	
HEPARIN	
RIVAROXABAN	
WARFARIN	

#### Anti-platelet medications

**Table:**

ABCIXIMAB	
ASPIRIN	
CLOPIDOGREL	
DIPYRIDAMOLE	
EPTIFIBATIDE	
PRASUGREL	
TICAGRELOR	
TICLOPIDINE	
TIROFIBAN	

#### Antihypertensive medications

**Table:**

AMLODIPINE	
ATENOLOL	
BENDROFLUAZIDE	
BISOPROLOL	
BUMETANIDE	
CANDESARTAN	
CAPTOPRIL	
CARVEDILOL	
CHLORTHALIDONE	
CLONIDINE	
DIAZOXIDE	
DILTIAZEM	
ENALAPRIL	
EPROSARTAN	
FELODIPINE	
FOSINOPRIL	
HYDRALAZINE	
HYDROCHLOROTHIAZIDE	
INDAPAMIDE	
IRBESARTAN	
LABETALOL	
LERCANIDIPINE	
LISINOPRIL	
LOSARTAN	
METHYLDOPA	
METOPROLOL	
MOXONIDINE	
NEBIVOLOL	
NIFEDIPINE	
NIMODIPINE	
OLMESARTAN	
OXPRENOLOL	
PERINDOPRIL	
PHENOXYBENZAMINE	
PINDOLOL	
PRAZOSIN	
PROPRANOLOL	
QUINAPRIL	
RAMIPRIL	
SOTALOL	
SPIRONOLACTONE	
TELMISARTAN	
TRANDOLAPRIL	
VALSARTAN	
VERAPAMIL	

#### Lipid lowering medications – fibrate

**Table:**

FENOFIBRATE	
GEMFIBROZIL	

#### Lipid lowering medications – statin

**Table:**

ATORVASTATIN	
FLUVASTATIN	
PRAVASTATIN	
ROSUVASTATIN	
SIMVASTATIN	

#### Lipid lowering medications – other

**Table:**

CHOLESTYRAMINE	
COLESTIPOL	
EZETIMIBE	
NICOTINIC ACID	

## Appendix 2

### Interview questions for stakeholders from general practices and Primary Health Network

**Table 4:** Interview questions for stakeholders in general practices

1. Why did their practice enrol?	
2. What happened in their practice during the project? What went well and what was difficult	
3. What members of the practice engaged with the project / tasks – who and why they were involved.	
4. Who set/s the priorities for doing tasks in the practice	
5. How did the practice work together as a team (or not) on the project?	
6. What could have been improved to assist them in participating in the project?	
7. Were they continuing to do QI work?	
8. Try to provide some details around the following	
a) Barriers and enablers to the implementation	
b) What worked well and what did not work well?	
c) What would they want to do differently if involved in a similar project in the future?	
d) What would they want done differently to assist in their participation in the future?	
e) Leadership in their practice	
f) Organisational QI Culture	
g) Funding Incentives to do this work	
h) Data- access and ability to use – are they doing anything now?	
i) Clinical Systems – have they done any work on this area in their practice?	
j) External support – how has the PHN or any other organisation assisted them in any way to do this work?

**Table 5:** Interview questions for stakeholders in Primary Health Networks

1. Outline their role at the PHN for Q Pulse (or similar QI projects)	
2. What was their experience of the project implementation – what worked and what was challenging	
3. What was their experience of QI project implementation more generally – what worked and what was challenging	
Then ask some more specific questions about the following areas:	
a) Barriers and enablers to the implementation	
b) What worked well and what did not work well?	
c) What would they want to do differently if involved in a similar project in the future?	
d) What would they want done differently to assist in their participation in the future?	
e) Leadership in the workplace	
f) Organisational QI Culture	
g) Incentives to do this work from Executive level / funding body	
h) Data- access and ability to use – are they doing anything now?	
i) Clinical Systems – have they done any work on this area in the PHN?	
j) External support – has any other organisation assisted the PHN in any way to do this work?	

## Abbreviations

BMI: Body mass index
BP: Blood pressure
CVD: Cardiovascular disease
GP: General practitioner
HDL: High-density lipoprotein
LDL: Low-density lipoprotein
PDSA: Plan-Do-Study-Act
PHN: Primary Health Network
QI: Quality improvement
QIC: Quality improvement collaboration
