# Supplementary material for: Findings and lessons learnt implementing a cardiovascular disease quality improvement program in Australian primary care: a mixed method evaluation
Source: BMC Health Serv Res. 2022 Jan 26;22:108. doi: 10.1186/s12913-021-07310-6 (PMC8790896; doi:10.1186/s12913-021-07310-6)
Supplement: Supplementary file 1 — Additional file 1: Supplementary Table 1. Baseline characteristics of included and excluded patients. Supplementary Table 2. Pre- and post-intervention characteristics of 4 sample practices. [file 12913_2021_7310_MOESM1_ESM.docx]

**Supplementary Table 1: Baseline characteristics of included and excluded patients**

|  | Bivariate |  | | | Univariate | | |  |
| --- | --- | --- | --- | --- | --- | --- | --- | --- |
|  | Study sample N= 19795 | Excluded sample  N= 17018 | | | OR (95% CI) of study sample compared to excluded sample^a^ | | | P-value |
| Age in years (mean, SD) | 63.6 (1.3) | 61.3 (1.1) | | | 0.99 (0.96-1.02) | | | 0.31 |
|  |  |  | | |  | | |  |
| **Gender** |  |  | | |  | | |  |
| Male | 44.7% (8822) | 46.9% (7972) | | | 0.91 (0.75-1.11) | | | 0.34 |
| Female | 55.3% (10936) | 53.1% (9039) | | | 1.00 | | |  |
|  |  |  | | |  | | |  |
| **Ethnicity** |  |  | | |  | | |  |
| Aboriginal or Torres Strait Islander | 0.6% (127) | 2.0% (341) | | | 0.37 (0.07-1.91) | | | 0.22 |
| Other | 99.4% (19668) | 98.0% (16677) | | | 1.00 | | |  |
|  |  |  | | |  | | |  |
| **Cardiovascular (CVD) risk** | | |  | | |  | |  |
| High CVD risk | 10.5% (2080) | 11.2% (1911) | | | 0.84 (0.61-1.15) | | | 0.25 |
| Established CVD | 12.2% (2407) | 9.4% (1594) | | | 1.19 (0.90-1.57) | | | 0.45 |
| Low/moderate risk | 41.4% (8191) | 44.8% (7622) | | | 1.00 | | |  |
| Complete risk factor data | 51.9% (10271) | 56.0% (9533) | | | 0.89 (0.42-1.89) | | | 0.88 |
|  |  |  | | |  | | |  |
| **Target blood pressure (BP)** | | |  | | |  | |  |
| Target BP achieved | 49.1% (9716) | 47.2% (8039) | | | 0.91 (0.63-1.33) | | | 0.61 |
|  |  |  | | |  | | |  |
| **Target blood lipids** |  |  | | |  | | |  |
| Total cholesterol < 4.0 mmol/L | 12.6% (2486) | | | 13.8% (2350) | | | 0.98 (0.72-1.33) | 0.88 |
| LDL cholesterol < 2.0 mmol/L | 15.4% (3045) | | | 23.8% (4057) | | | 0.63 (0.35-1.14) | 0.12 |
| HDL cholesterol > 1.0 mmol/L | 53.2% (10529) | | | 53.2% (9061) | | | 2.25 (1.36-3.73) | <0.01 |
|  |  |  | | |  | | |  |

A Univariate analyses were adjusted for gender, age and ethnicity.

**Supplementary Table 2: Pre- and post-intervention characteristics of 4 sample practices**

|  | **Practice 1** | | **Practice 2** | | **Practice 3** | | **Practice 4** | |
| --- | --- | --- | --- | --- | --- | --- | --- | --- |
|  | **Pre** | **Post** | **Pre** | **Post** | **Pre** | **Post** | **Pre** | **Post** |
| In scope pts | 2053 | 1975 | 736 | 712 | 187 | 174 | 2019 | 2165 |
|  |  |  |  |  |  |  |  |  |
| **Risk factor assessment** | |  |  |  |  |  |  |  |
| Blood pressure^a^ | 46.3 (951) | 54.0* (1066) | 92.0 (677) | 93.7 (667) | 98.9 (185) | 97.7 (170) | 89.6 (1809) | 87.6 (1897) |
| Blood lipids^b^ | 62.2 (1276) | 73.4* (1444) | 91.6 (674) | 94.2 (671) | 88.8 (166) | 92.0 (160) | 71.4 (1442) | 70.6 (1528) |
| BMI^b^ | 10.5 (115) | 18.0 (320) | 81.8 (602) | 91.7* (653) | 94.7 (177) | 96.6 (168) | 84.2 (1699) | 81.8 (1771) |
| Waist circumference^b^ | 0.7 (15) | 18.5* (366) | 32.2 (237) | 69.8* (497) | 6.4 (12) | 25.3* (143) | 57.2 (1154) | 51.8 (1121) |
| Smoking status^b^ | 71.2 (1461) | 69.8 (1221) | 73.8 (543) | 73.2 (521) | 63.2 (119) | 65.9 (114) | 72.1 (1455) | 71.8 (1555) |
|  |  |  |  |  |  |  |  |  |
| **BMI** |  |  |  |  |  |  |  |  |
| Underweight | 0.5 (11) | 0.6 (12) | 4.5 (33) | 4.6 (33) | 7.0 (13) | 6.3 (11) | 5.2 (105) | 5.2 (117) |
| Healthy weight | 4.3 (88) | 5.4 (106) | 25.4 (187) | 29.1 (207) | 27.8 (52) | 25.3 (44) | 33.3 (672) | 32.7 (708) |
| Overweight/obese | 7.9 (163) | 9.8 (193) | 34.4 (253) | 33.8 (241) | 32.6 (61) | 36.8 (64) | 33.7 (681) | 33.4 (724) |
| Not assessed/missing | 87.2 (1791) | 84.3 (1664) | 35.7 (263) | 32.4 (231) | 32.6 (61) | 31.6 (55) | 27.8 (561) | 28.5 (616) |
|  |  |  |  |  |  |  |  |  |
| **Waist circumference** | |  |  |  |  |  |  |  |
| Normal | 0.5 (10) | 0.7 (13) | 5.2 (38) | 12.9 (92) | 15.0 (28) | 15.5 (27) | 22.4 (453) | 22.6 (490) |
| At risk^c^ | 1.1 (23) | 2.0 (40) | 47.7 (351) | 17.1 (122) | 48.7 (91) | 17.8 (31) | 23.3 (471) | 21.0 (454) |
|  |  |  |  |  |  |  |  |  |
| **Smoking status** |  |  |  |  |  |  |  |  |
| Never smoker | 48.6 (997) | 47.4 (937) | 57.2 (421) | 57.0 (406) | 61.5 (115) | 60.3 (105) | 48.1 (971) | 49.2 (1066) |
| Current smoker | 11.1 (228) | 11.9 (235) | 26.8 (197) | 29.2 (208) | 19.3 (36) | 20.7 (36) | 38.7 (781) | 37.3 (808) |
| Ex-smoker | 18.2 (373) | 17.9 (353) | 11.7 (86) | 11.0 (78) | 17.1 (32) | 16.7 (29) | 12.7 (257) | 12.7 (275) |
| Missing | 22.2 (453) | 22.8 (450) | 4.3 (32) | 2.8 (20) | 2.1 (4) | 2.3 (4) | 0.5 (10) | 0.7 (16) |
|  |  |  |  |  |  |  |  |  |
| **CVD risk estimation** | |  |  |  |  |  |  |  |
| Low/moderate | 12.8 (262) | 30.9 (610) | 59.0 (434) | 63.5 (452) | 60.4 (113) | 54.6 (95) | 64.7 (1306) | 66.5 (1440) |
| High | 3.7 (76) | 10.3 (203) | 14.3 (105) | 14.2 (101) | 18.2 (34) | 17.8 (31) | 10.5 (212) | 10.5 (227) |
| Established CVD | 5.2 (106) | 5.7 (113) | 16.6 (122) | 16.7 (119) | 13.9 (26) | 20.7 (36) | 8.8 (178) | 10.3 (224) |
| Insufficient data to assess | 83.5 (1715) | 58.8* (1162) | 26.8 (197) | 22.3 (159) | 21.4 (40) | 27.6 (48) | 24.8 (501) | 23.0 (498) |
|  |  |  |  |  |  |  |  |  |
| **CVD targets achieved** | |  |  |  |  |  |  |  |
| Blood pressure | 78.5 (747) | 67.5 (720) | 87.1 (590) | 83.1 (554) | 58.4 (108) | 49.4 (84) | 72.8 (1317) | 73.4 (1393) |
| Total cholesterol | 13.5 (173) | 15.6 (226) | 32.5 (219) | 30.6 (205) | 21.1 (35) | 20.6 (33) | 14.3 (206) | 15.6 (239) |
| LDL cholesterol | 26.9 (343) | 27.1 (392) | 35.8 (241) | 31.3 (210) | 21.7 (36) | 24.4 (39) | 17.8 (257) | 19.1 (292) |
| HDL cholesterol | 70.1 (384) | 73.2 (1062) | 89.2 (601) | 90.3 (606) | 92.2 (153) | 87.5 (140) | 89.9 (1292) | 90.3 (1380) |
|  |  |  |  |  |  |  |  |  |
| **Prescribed CVD risk-reducing medications** | | |  |  |  |  |  |  |
| Prescribed recommended risk-reducing medication^d^ | 51.3 (39) | 53.7 (109) | 53.3 (56) | 51.5 (52) | 47.1 (16) | 54.8 (17) | 46.7 (99) | 46.3 (105) |

A Assessed in the past 12 months.

B Assessed in the past 2 years.

C ‘At risk’ waist circumference >94cm for males and > 80cm for females.

D Guideline-recommended treatment was defined as (1) For high-risk patients the prescription of a BP-lowering medication and a statin; [5] for patients with established CVD, prescription of a BP-lowering medication, a statin and either an antiplatelet or an anticoagulant agent (see Appendix A for specific medications).

* Statistically significant (p<0.05) between baseline and follow-up.
